# Metabolomics Fingerprint Induced by the Intranigral Inoculation of Exogenous Human Alpha-Synuclein Oligomers in a Rat Model of Parkinson’s Disease

**DOI:** 10.3390/ijms21186745

**Published:** 2020-09-14

**Authors:** Federica Murgia, Luigi Atzori, Ezio Carboni, Maria Laura Santoru, Aran Hendren, Augusta Pisanu, Pierluigi Caboni, Laura Boi, Giuliana Fusco, Anna R. Carta

**Affiliations:** 1 Clinical Metabolomics Unit, Department of Biomedical Sciences, University of Cagliari, Cittadella Universitaria, Monserrato, 09042 Cagliari, Italy; latzori@unica.it (L.A.); marialaurasantoru@gmail.com (M.L.S.); a.hendren@studenti.unica.it (A.H.); 2 Neuroscience Section, Department of Biomedical Sciences, University of Cagliari, Cittadella Universitaria, Monserrato, 09042 Cagliari, Italy; ecarboni@unica.it (E.C.); lauraboi@unica.it (L.B.); 3 Faculty of Health and Medical Sciences, University of Surrey, London GU2 7XH, UK; 4 CNR Institute of Neuroscience, Monserrato, 09042 Cagliari, Italy; Augusta.Pisanu@in.cnr.it; 5 Department of Life and Environmental Sciences, University of Cagliari, Cittadella Universitaria, Monserrato, 09042 Cagliari, Italy; caboni@unica.it; 6 Department of Chemistry, Centre for Misfolding Diseases, University of Cambridge, Cambridge CB2 1EW, UK; gf203@cam.ac.uk

**Keywords:** α-synuclein oligomers, Parkinson model, metabolomics, mesencephalic tissue, serum, gas chromatography mass spectrometry, biomarkers

## Abstract

Parkinson’s disease (PD) is considered a synucleinopathy because of the intraneuronal accumulation of aggregated α-synuclein (αSyn). Recent evidence points to soluble αSyn-oligomers (αSynO) as the main cytotoxic species responsible for cell death. Given the pivotal role of αSyn in PD, αSyn-based models are crucial for the investigation of toxic mechanisms and the identification of new therapeutic targets in PD. By using a metabolomics approach, we evaluated the metabolic profile of brain and serum samples of rats infused unilaterally with preformed human αSynOs (HαSynOs), or vehicle, into the substantia nigra pars compacta (SNpc). Three months postinfusion, the striatum was dissected for striatal dopamine (DA) measurements via High Pressure Liquid Chromatography (HPLC) analysis and mesencephalon and serum samples were collected for the evaluation of metabolite content via gas chromatography mass spectrometry analysis. Multivariate, univariate and correlation statistics were applied. A 40% decrease of DA content was measured in the HαSynO-infused striatum as compared to the contralateral and the vehicle-infused striata. Decreased levels of dehydroascorbic acid, myo-inositol, and glycine, and increased levels of threonine, were found in the mesencephalon, while increased contents of fructose and mannose, and a decrease in glycine and urea, were found in the serum of HαSynO-infused rats. The significant correlation between DA and metabolite content indicated that metabolic variations reflected the nigrostriatal degeneration. Collectively, the metabolomic fingerprint of HαSynO-infused rats points to an increase of oxidative stress markers, in line with PD neuropathology, and provides hints for potential biomarkers of PD.

## 1. Introduction

Abnormal aggregation of specific pathogenic proteins within the central nervous system has been recognized as a fundamental feature of several neurodegenerative disorders, including Parkinson’s disease (PD) [1]. PD is considered a synucleinopathy due to the abnormal accumulation of misfolded, and often largely phosphorylated, α-synuclein (αSyn) in cells of the central nervous system (CNS) [2]. While the pathological role of aggregated αSyn in PD is generally acknowledged, increased levels of soluble intermediates of the aggregation process, such as small size oligomers (αSynO), have been described in biological fluids of PD patients, raising questions as to their toxic role in the disease [3,4]. In recent years, several preclinical studies have suggested that αSynOs are the most toxic αSyn species against neurons [5,6,7].

α-Syn neurotoxicity has defined the rationale for αSyn-based preclinical models of PD [8,9] and, in recent years, the intracerebral inoculation of preformed αSyn fibrils and oligomers has been proposed as a significant disease model [10,11,12,13,14,15]. Lately, preformed αSyn oligomers of human origin (HαSynO) have been produced and characterized for their in vitro and in vivo toxicity in neurons [7,16]. These studies demonstrated that these short oligomers hold a higher neurotoxicity compared to the monomeric species of the protein. Furthermore, we found that the intracerebral inoculation of these HαSynOs induced a gradual nigrostriatal dopaminergic loss associated with motor and cognitive impairment, the deposition of phosphorylated αSyn in neurons and microglia of dopaminergic areas, an early and persistent neuroinflammatory response and mitochondrial abnormalities, suggesting that it is a valuable preclinical model of PD that reproduces the cardinal features of the pathology [unpublished results].

Modeling the αSyn-related neuropathology may contribute to the investigation of toxic mechanisms in PD and may drive the identification of new therapeutic targets. Moreover, preclinical models that replicate neuropathological features of PD offer an essential tool for validating the use of disease biomarkers to monitor the evolution and the therapeutic response of PD to new treatments. The search for disease biomarkers has made significant progresses in past years, leading to the identification of several clinically validated biomarkers [17,18,19,20].

In this context, a nontargeted approach with metabolomics analysis provides a powerful tool to unveil neuropathological mechanisms and to investigate potential disease biomarkers that may differentiate complex diseases, such as neurodegenerative disorders, from nondiseased conditions. Metabolomics starts with the axiom that the disease state and its therapeutic response, together with lifestyle factors, can be reflected by changes in metabolite concentrations [21]. Interestingly, metabolomics allows the unbiased simultaneous evaluation of multiple biomolecules in a single experimental sample, usually through the use of nuclear magnetic resonance spectroscopy [22,23] and/or mass spectrometry (MS) platforms [24,25]. In PD, several studies have investigated the metabolic profile of cerebrospinal fluid (CSF) and serum of PD patients to identify possible disease biomarkers [26,27].

The aim of the present work was to evaluate the metabolic profile of brain tissue and serum samples of rats inoculated with preformed HαSynOs into the substantia nigra pars compacta (SNpc) [10,11]. Given the human origin of the inoculated oligomers, and since this model reproduces neuropathological mechanisms of PD, we investigated whether it may offer a tool to model the metabolic fingerprint of PD.

## 2. Results

Gas chromatography mass spectrometry (GC-MS) analysis performed on tissue from the right and left mesencephalon and on serum from rats (*n* = 7) inoculated with HαSynOs into the left SNpc (Oligo class, Right (R) and Left (L), respectively) or with vehicle (*n* = 7) (Veh class, Right (R) and Left (L), respectively) showed significant metabolic changes in the inoculated rats compared with vehicles, and a correlation with striatal dopamine (DA) content. Serum samples were collected to detect any peripheral metabolic alteration resulting from the intracerebral infusion of HαSynOs. Through GC-MS analysis, we identified 25 and 27 metabolites in the mesencephalon and serum samples, respectively, including sugars, amino acids, fatty acids and biogenic amines.

### 2.1. Striatal DA and DOPAC Assessment

**Figure 1:**
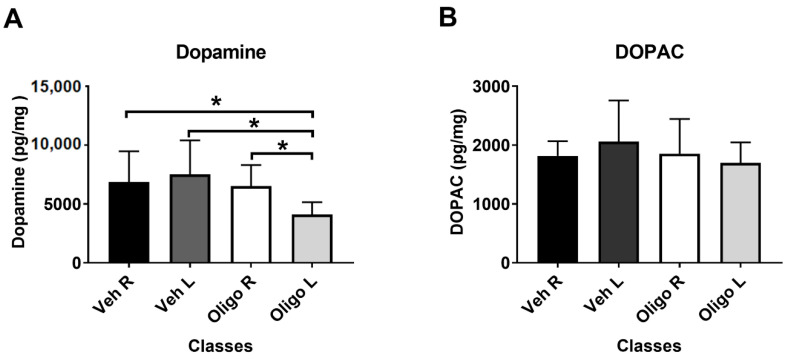
Striatal content of dopamine (DA) (**A**) and 3,4-Dihydroxyphenylacetic acid (DOPAC) (**B**). DA and DOPAC levels (pg/mg fresh tissue) were measured in the left (L) and right ^®^ striata of rats infused with human α-synuclein oligomers (HαSynOs) or vehicle, three months after inoculation. Data are expressed as the mean and the standard deviation for each class. * *p* < 0.05).

Figure 1A shows that the inoculation of HαSynOs into the left SNpc caused a significant reduction of DA tissue levels in the ipsilateral striatum, compared with the contralateral striatum and with the left striatum of vehicle-infused rats. One way-ANOVA of the results showed a significant effect of treatment (F_3,23_ = 4.11). Post hoc analysis (Tukey test) indicated that DA tissue levels in the left striatum of HαSynOs-infused rats were significantly lower with respect to those assessed in both the L and R striata of vehicle inoculated rats (*p* < 0.05) and with respect to the contralateral striatum. Figure 1B shows that the infusion of HαSynOs into the left SNpc did not cause significant changes of 3,4-Dihydroxyphenylacetic acid (DOPAC) levels in the ipsilateral striatum when compared with the contralateral striatum or with the L and R striatum of vehicle-infused rats. Based on these results, striatal DA concentration only was considered for statistical correlation with the metabolic profile. 

### 2.2. Multivariate Analysis

**Figure 2:**
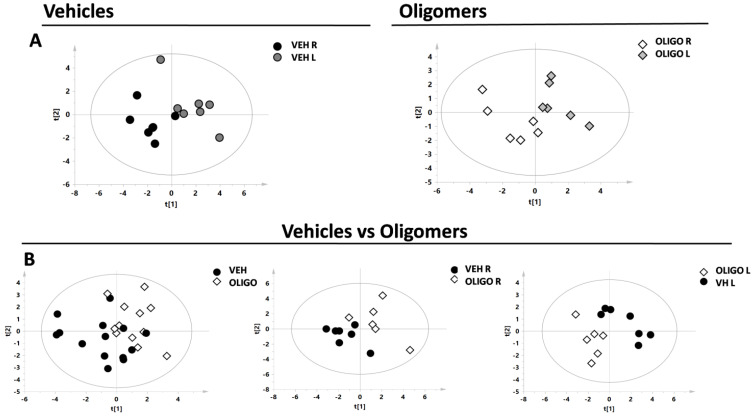
Score plot of the supervised models of R and L mesencephalon. The scores of the samples represent the result of the association/covariance between the Y-variables (treated or not-treated with oligomers rats) and metabolites concentrations as predictors (X-variables). (**A**) Partial least square discriminant analysis (PLS-DA) models comparing the R and L mesencephalon from Vehicle (respectively black and dark grey circles) or Oligo (respectively white and light grey diamonds) classes; (**B**) PLS-DA models comparing the mesencephalon of the Vehicle class (black circles) and Oligo class (white diamonds).

Firstly, the metabolic changes in the mesencephalon were investigated. Principal component analysis (PCA) and the resultant score plot, together with the T^2^ Hotelling test, indicated the presence of one outlier belonging to the oligo class (unpublished results). A supervised model (partial least square discriminant analysis, PLS-DA) was first performed by analysing the R and L mesencephalon from rats belonging to the same class (vehicle or oligomers, respectively, Figure 2A). Subsequently, the PLS-DA model was performed on data from the mesencephalon of the different classes, vehicle and oligomers (Figure 2B). In Figure 2B, the plot resulting from the comparison of samples from all classes (including R and L mesencephalon) is shown on the left, followed by plots resulting from the comparisons of the R or L mesencephalon, respectively, (central and right panels in Figure 2B). The statistical parameters, as indicated in Table 1, demonstrated good separation of the Oligo R vs. Oligo L, and Vehicle L vs. Oligo L.

**Figure 3:**
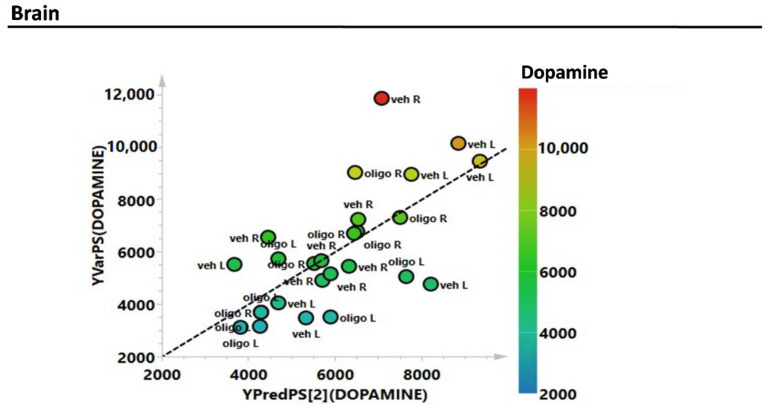
Partial least squares (PLS) correlation analysis of the complete metabolic profile of mesencephalon samples with striatal levels of DA (expressed as pg/mg tissue).

Subsequently, the estimation of the correlations of the complete metabolic profile of L and R mesencephalon with striatal DA were evaluated through the partial least squares (PLS) regression analysis (Figure 3). The correlation between the complete metabolic profile of the L and R hemispheres with the striatal DA levels measured in the same hemispheres showed an R^2^ = 0.6.

**Figure 4:**
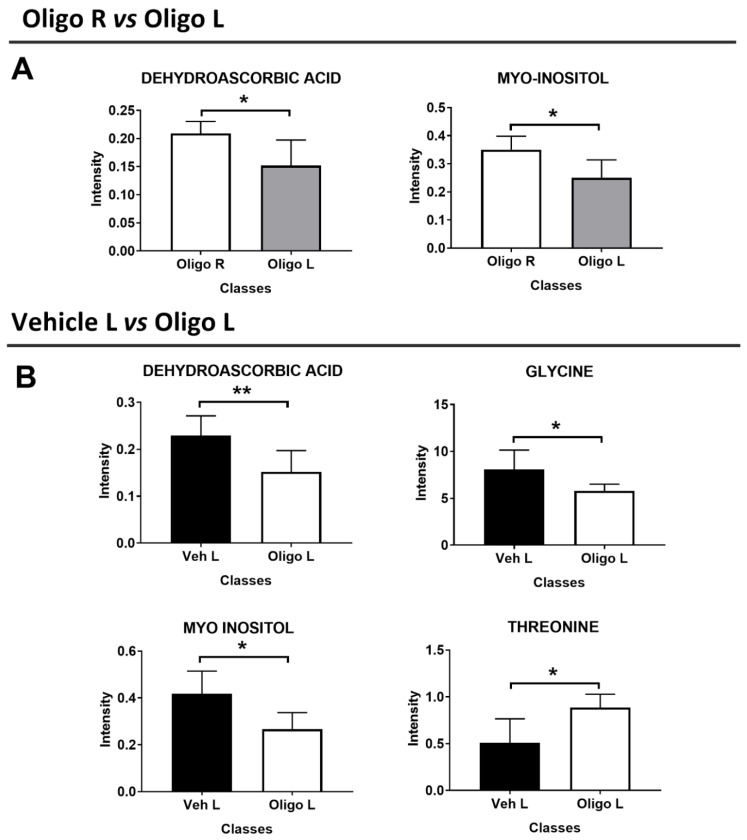
Discriminant metabolites resulting from the multivariate analysis of the mesencephalon between Oligo R vs. Oligo L (**A**), and Veh L vs. Oligo L (**B**). U-Mann Whitney analysis was performed to evaluate the statistical significance. * = *p*-value < 0.05; ** = *p*-value < 0.001.

Based on the statistical parameters of the multivariate PLS-DA models, the models Oligo R vs. Oligo L, and Veh L vs. Oligo L were considered. Through the analysis of the loadings plot, it was possible to identify the variables responsible for the separation of the classes. For the model Oligo R vs. Oligo L, the discriminant metabolites were dehydroascorbic acid (DHAA) and myo-inositol. These metabolites were tested with the U-Mann Whitney test to evaluate the statistical significance, and the *p*-values were < 0.05. (Figure 4A).

Similarly, the metabolites responsible for the separation between Veh L vs. Oligo L were identified and were tested through the U-Mann Whitney test. Changes in DHAA, glycine, myo-inositol, and threonine represented the specific pattern of the L part of the mesencephalon infused with oligomers and the *p*-values were < 0.05 (Figure 4B). Moreover, Holm-Bonferroni correction was applied. All the statistical results are reported in Table 2.

**Figure 5:**
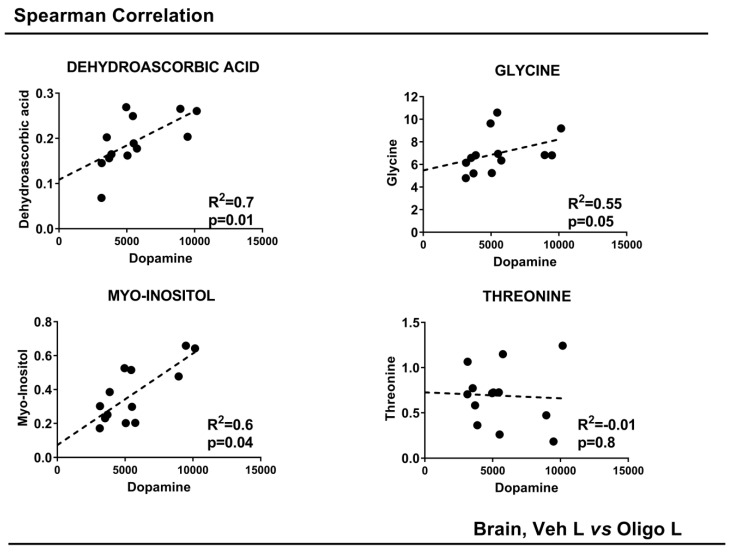
Spearman Correlation of striatal DA concentrations with the concentration of metabolites in the mesencephalon. *p*-value < 0.05.

The concentrations of DA were correlated with the concentrations of the single varying metabolites with a *p*-value < 0.05: DHAA, glycine, myo-inositol and threonine. To achieve this, a Spearman correlation was performed. The results are shown in Figure 5. DHAA, glycine and myo-inositol showed a good linear positive correlation. Threonine did not show any correlation. All the parameters are reported in Table 2.

**Figure 6:**
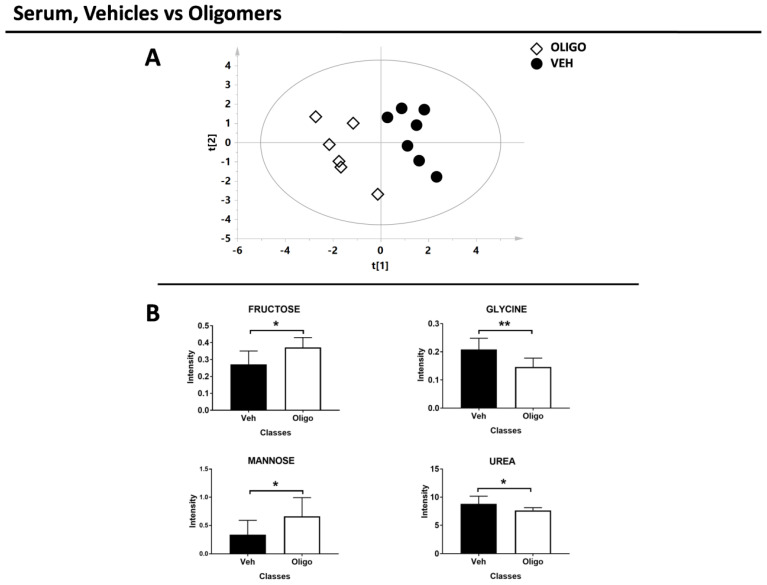
Results of the multivariate and univariate analyses of the serum sample. (**A**) Score plot of the rats infused with oligomers (white diamonds) and vehicles (black circles). (**B**) Bar graphs of the discriminant metabolites. U-Mann Whitney test was performed and these metabolites exhibit *p*-value < 0.05. * = *p*-value < 0.05; ** = *p*-value < 0.001

In the second part of the analysis, the serum samples of rats infused with HαSynOs were compared with the serum samples from vehicle-infused rats. The multivariate supervised analysis underlined a good clustering of the two classes. The score plot is reported in Figure 6A (R^2^X = 0.38, R^2^Y = 0.89, Q^2^ = 0.56, *p* = 0.01).

**Figure 7:**
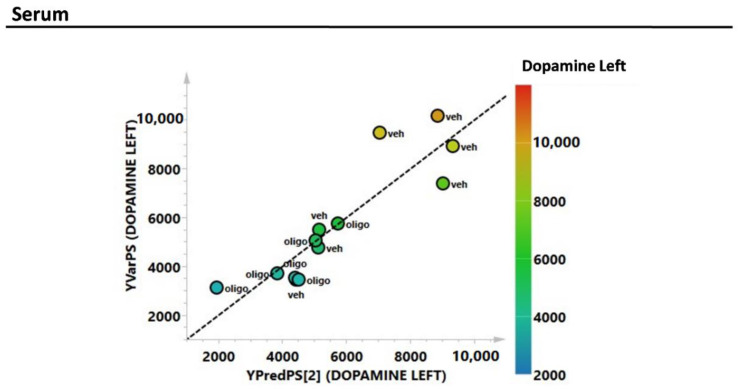
PLS correlation analysis of the complete metabolic profile of the serum samples with the level of the L DA (expressed as pg/mg tissue).

As with the mesencephalon tissue analysis, the correlation of the complete metabolic profile of the serum samples from rats infused with oligomers or vehicles with L striatal DA was evaluated through PLS analysis (Figure 7). The correlation between the DA in the L hemisphere and the metabolic profile of the serum showed an R^2^ = 0.78.

The serum metabolites responsible for the PLS-DA separation between rats infused with oligomers or vehicle were identified and tested through the U-Mann Whitney test. After the test, fructose, glycine, mannose, and urea resulted the discriminant metabolites (Figure 6B). The statistical parameters are reported in Table 3 without or with Holm-Bonferroni correction.

## 3. Discussion

The aim of this study was to investigate the metabolic fingerprint in the brain and serum of a new PD rat model induced by the inoculation of exogenous HαSynOs. Protein misfolding in the central nervous system is a key pathological mechanism in PD, which is currently ascribed to the family of synucleinopathies, characterized by the accumulation of misfolded aggregates of αSyn fibrils in neuronal and non-neuronal brain cells [1,28]. αSyn aggregation is a heterogeneous process generating a variety of intermediate structures [5,29,30]. Hence, although fibrillar aggregates of αSyn within Lewy bodies are a hallmark of PD, recent in vitro and preclinical evidence indicates that small soluble oligomers are the most toxic species towards neurons [5,31,32,33,34,35,36,37]. Moreover, recent studies have demonstrated that the toxicity of the αSyn oligomer is also structure-specific and species-dependent [5,7,15,16].

The intracerebral inoculation of preformed αSyn fibrils and oligomers is increasingly used as a preclinical model of PD [10,11,12,13,14,15]. Indeed, the presence of soluble oligomers in affected brain areas [38] and biological fluids of PD patients [3,4], suggested the pathological role of oligomeric species of αSyn. Here, we inoculated into the SNpc highly purified oligomers of αSyn composed of recombinant human αSyn. These oligomers have been shown to possess elevated homogeneity for size distribution and structure, as shown in previous characterizations based on analytical ultracentrifugation (AUC), nuclear magnetic resonance (NMR), atomic force microscopy (AFM) and Fourier transform infrared (FT-IR) [7,30]. Furthermore, in vitro and in vivo studies on these oligomers kinetically trapped in a toxic conformation, have evidenced the effect of specific antibodies in reducing the toxicity and cell damage of these aggregates [7,16]. Oligomeric species of αSyn exert their toxic activity in several cellular processes [39], including mitochondrial function, microtubule polymerisation, calcium signalling, protein degradation and interaction with glial cells to promote neuroinflammation [40]. Decreased efficiency of the aforementioned pathways increases the burden of cellular stress, eventually leading to oxidative stress, neuroinflammation and neurotoxicity [41] in brain areas affected by the disease, and to the degeneration of nigrostriatal neurons.

We have recently found that the intranigral inoculation of HαSynOs, but not the inoculation of the same amount of vehicle, generated most neuropathological and symptomatic features of PD, including the slow degeneration of nigrostriatal neurons reflected by decreased striatal DA and motor impairment, mitochondrial damage and neuroinflammation [unpublished observation]. Therefore, we conclude that the altered metabolic profile described in the present study was causally linked to the intracerebral inoculation of these HαSynOs. In the present study, we further characterised this HαSynO-based model, by analysing and comparing, via GC-MS, the specific metabolic pattern of the mesencephalic tissue and the serum of oligo and vehicle classes. Furthermore, metabolites responsible for discrimination in the mesencephalon and serum were defined. Decreased levels of DHAA, myo-inositol and glycine, and increased levels of threonine, were found in the brain, while increased concentrations of fructose and mannose, and a decrease in glycine and urea, were found in the serum. The striatal tissue from the same rats was analyzed for DA and DOPAC levels, revealing a 40% decrease of DA content, reflecting the nigrostriatal degeneration. Importantly, the complete metabolic profile, as well as several discriminant metabolites in brain and serum, displayed good correlations with levels of striatal DA, suggesting that metabolic changes in brain and serum were causally correlated with the HαSynOs-induced nigrostriatal degeneration. Tissue analysis also showed a not significant reduction in DOPAC concentration in the striatum ipsilateral to the HαSynO injection. Although DOPAC is the main DA metabolite, its tissue levels also reflect the turnover of DA. Thus, in the present study the reduced synaptic concentration of DA may have stimulated DA turnover attenuating the decrease of DOPAC compared with that of DA resulting in an increased DOPAC/DA ratio. PD is now recognized as a systemic disorder, affecting several brain areas but also the peripheral nervous system and peripheral functions, as well as the immune system as a whole. Accumulation of αSyn may start in the gut and then propagate to the CNS via the sympathetic nervous system, the vagus and the glossopharyngeal nerves [42,43]. Moreover, neuronal damage within the CNS and mitochondrial disruption may trigger systemic inflammation, while brain-derived exosomes carrying damage-associated molecules can pass the blood-brain barrier and are found into the plasma [44]. Results of the present study suggest that HαSynOs-induced damage is not confined to the CNS but may trigger systemic dysfunctions shown by altered serum metabolites. Notably, this supports the concept of changes in serum metabolites correlated with striatal DA levels.

Several studies have investigated the metabolomic signature and amino acids profile of parkinsonian patients in the search of biomarkers for early diagnosis or therapeutic response, revealing a clear separation of subjects according to both measures [17,45]. Remarkably, the discriminant metabolites induced by HαSynOs-inoculation in our rat model were also demonstrated in de novo parkinsonian patients. Similar to metabolomic changes observed in the present study, the investigation of the CSF fluid via metabolomic profiling [26] or serum via amino acid profiling [46] revealed decreased levels of DHAA, glycine and urea, and increased levels of threonine, fructose, and mannose, suggesting that HαSynOs largely reproduced the metabolic profile of the human disease.

These findings support the inoculation of HαSynOs as a valid preclinical model of PD, which adds to previously collected evidence of neuropathological PD-like features of the model. Moreover, since oligomers were obtained via the aggregation of human αSyn, the present study supports the pivotal role of oligomeric species of αSyn in the human neuropathology of PD.

Metabolic changes observed both in the brain tissue and in the serum point to a burden of the oxidative stress response as a result of cellular toxicity of HαSynOs. DHAA is the oxidized form of the antioxidant ascorbic acid [47], and metabolomic analysis of mesencephalic tissue revealed a decrease in the oligo class compared with the vehicle class (*p* = 0.004). The Spearman correlation revealed a high degree of rank correlation between DHAA and striatal DA concentration (R^2^ = 0.77, *p* = 0.009). DHAA is the blood-brain barrier transportable form of ascorbic acid [48] and is converted to its active form within the brain. Ascorbic acid plays a fundamental role in neurons, specifically in mitochondria, where oxidative phosphorylation is the main source of free radical [47] generation. As an antioxidant, ascorbic acid protects the mitochondrial genome and membranes from oxidative damage. The present results suggest that the overload of oxidative stress caused by HαSynO neurotoxicity may account for the depletion of ascorbic acid in the brain. Previous studies have shown that αSyn overexpression inhibits complex I [49,50,51] and disrupts mitochondrial membrane integrity, leading to increased ROS production [52,53]. Moreover, mitochondrial damage has been specifically related to αSyn oligomer toxicity [50,54,55,56,57].

Increased levels of fructose and mannose in the serum of HαSynO-infused rats may similarly reflect a burden of oxidative stress responses. Fructose and mannose are highly implicated in protein glycation. In PD, glycation end-products of αSyn colocalize in Lewy bodies of the substantia nigra [58]. Interestingly, glycated αSyn undergoes oligomerization more easily, while glycated oligomers slow the fibril formation process [59,60]. Glycation is boosted by oxidative stress and leads to increased production of ROS, being itself an oxidative stress-promoting factor. In line with the present results, other markers of oxidative stress, such as malondialdehyde, oxidized glutathione and 8-hydroxydeoxyguanosine, together with low reduced-glutathione, have been described in the plasma of PD patients [61,62,63,64].

Myo-inositol was found at a lower concentration in the mesencephalon of HαSynO-injected rats compared with vehicle-injected rats (*p* = 0.04), suggesting that reduced levels resulted from HαSynO-induced neuropathology. Besides being a component of phosphatidylinositol in biological membranes, free myo-inositol has a key role in multiple cellular processes, such as maintenance of membrane potential, ion channel permeability, cytoskeletal remodeling [65] and stress response. Changes in myo-inositol concentration may, therefore, reflect impaired neuronal membrane stability caused by HαSynO-induced neuronal damage, and represent an additional biomarker of oxidative stress.

Interestingly, we found altered amino acids levels in the serum of HαSynO-injected rats, in line with reports on the serum of PD patients. Specifically, lower levels of glycine, both in the brain and serum, and elevated content of threonine in the brain were found. A recent study reported lower levels of specific amino acids, including glycine, in the serum of parkinsonian patients [46]. Moreover, threonine concentrations positively correlated with disease duration in PD [45]. Amino acids serve multiple functions and are precursors for the synthesis of a plethora of molecules from ATP to hormones and nucleic acids. In the brain, amino acids are profoundly involved in neurotransmission and affect brain functions. For instance, impaired glycine transmission was related to REM (Rapid Eye Movement) sleep behavior disorder [66], and changes in serum amino acids were correlated with PD symptoms [46]. Glycine is also used in glutathione synthesis, along with glutamate and cysteine [67]. Low glycine levels may, therefore, reflect the increased glutathione demand and consumption to face oxidative stress in PD [68,69,70]. Considering the altered level of urea that we found in the serum of the oligo class, as the possible role of urea decrease in Parkinson’s disease is still unknown we cannot explain the urea decrease observed in our experimental model.

## 4. Materials and Methods

### 4.1. Production of Recombinant H-αSyn

Recombinant HαSyn was purified in *E. coli* using plasmid pT7-7 encoding for the protein as previously described [7].

The expression was induced with 1 mM IPTG at 37 °C for 4 h. The cell lysate was centrifuged at 22,000× *g* (Beckman Coulter, Brea, CA, USA) for 30 min and the supernatant was then heated for 20 min at 70 °C. After centrifugation at 22,000× *g*, two steps of precipitation and centrifugation were employed and in particular 10 mg·mL^−1^ streptomycin sulfate was added to the supernatant for DNA precipitation and, subsequently, 360 mg·mL^−1^ ammonium sulfate was added to the supernatant to precipitate the recombinant HαSyn. The obtained pellet was resuspended in 25 mM Tris-HCl, pH 7.7 and, after dialysis against the same buffer, loaded onto an anion exchange column (26/10 Q sepharose high performance, GE Healthcare, Little Chalfont, UK) to be eluted with a 0–1 M NaCl step gradient. Further purification was obtained with size exclusion chromatography (Hiload 26/60 Superdex 75 preparation grade, GE Healthcare). The purity of the sample was analyzed by SDS-PAGE and the protein concentration was determined from the absorbance at 275 nm using an extinction coefficient of 5600 M^−1^·cm^−1^.

### 4.2. Purification of HαSynO

Toxic oligomeric samples were prepared from purified recombinant HαSyn as previously described [7]. Lyophilized protein was resuspended in PBS buffer at a pH of 7.4 and a concentration of 12 mg·mL^−1^, then passed through a 0.22 μm cut off filter before incubation at 37 °C for 24 h without agitation. Residual fibrillar species were removed by ultracentrifugation for 1 h at 288,000× *g* and excess of monomers were removed using several filtration steps with 100 kDa cutoff membranes. Samples of the toxic HαSyn oligomers prepared in this manner are stable for many days at room temperature, but in this study were used within two days of their production.

### 4.3. Animals and Stereotaxic Surgery

Male Sprague Dawley rats weighting 275–300 g were deeply anesthetized with Fentanyl (3 mg/Kg). HαsynOs were kept under gentle shaking at room temperature (RT) until they were infused into the left SNpc with 5 μL of HαSynOs (0.5 mg/mL, *n* = 7) or vehicle (*n* = 7) (coordinates from Bregma, according to the atlas of Paxinos and Watson, were as follows: anteroposterior: −5.4; mediolateral: −1.9; dorsoventral: −7.2) via a stainless-steel cannula, at the infusion rate of 1 μL/min. The cannula was left in place for additional 5 min after infusion and then slowly withdrawn. Three months after surgery, animals were deeply anesthetized, sacrificed by decapitation, and blood samples were collected. After brain removal, the striatum and the mesencephalon from the side homolateral as well as contralateral to HαsynOs injection were dissected on ice, collected separately and stored at −80° for subsequent analysis. All experimental procedures complied with the ARRIVE guidelines and were in accordance with the guidelines and protocols approved by the European Community (2010/63UE L 276 20/10/2010). Experiments were approved by OPBA (animal care committee) at the University of Cagliari and by the Italian Ministry of Health (Aut. 766-2020/PR).

### 4.4. Sample Preparation

Mesencephalic tissue: 50 mg of tissue were suspended with 600 μL of methanol. Tissue was homogenized for five minutes with a TissueLyser instrument (Tissuelyser 2, Quiagen, Hilden, Germany) and subsequently 600 μL of chloroform and 400 μL of Milli-Q water were added. After 30 min of sonication, samples were kept at −20 °C for 20 min and then centrifuged at 8600× *g* for 10 min at 4 °C. The hydrophilic phase was collected for the instrumental analysis. The water-phase was concentrated overnight using a speed vacuum centrifuge (Eppendorf concentrator plus, Eppendorf AG, Hamburg, Germany).

Serum: serum samples were thawed and firstly centrifuged at 1200× *g* for 15 min at room temperature (RT). An aliquot of 400 μL was mixed to 1200 μL of a chloroform/methanol 1:1 plus 175 μL of distilled water. The samples were vortexed and centrifuged for 30 min at 1700× *g* at RT. Two phases were obtained, the hydrophilic and hydrophobic phases. The hydrophilic phase was concentrated overnight using a speed vacuum centrifuge (Eppendorf concentrator plus, Eppendorf AG, Hamburg, Germany)

### 4.5. GC-MS Derivatization

Derivatization was performed by adding 25 μL and 100 μL, respectively, for mesencephalic tissue and serum sample, of methoxyamine hydrochloride in pyridine solution (10 mg/mL) (Sigma-Aldrich, St. Louis, MO, USA) to dried brain samples at 70 °C. After 1 h, 50 and 100 μL, respectively, for mesencephalic tissue and serum sample of N-Methyl-N-(trimethylsilyl)-trifluoroacetamide (MSTFA, Sigma-Aldrich, St. Louis, MO, USA) were added and the samples were left at room temperature for 1 h. Samples were then diluted in hexane (50 μL for the mesencephalic tissue and 600 μL for the serum sample) with an internal standard (undecane at 25 ppm). For the serum samples, diluted samples were then filtered (PTFE 0.45 μm) and transferred into glass vials. As before, sample blanks were made to avoid noise caused by the laboratory instruments or by the chemicals used for the preparation, by following the same procedure.

### 4.6. GC-MS Analysis and Data Processing

The derivatized sample (1 μL) was injected in splitless mode into a 7890A gas chromatograph coupled with a 5975C Network mass spectrometer (Agilent Technologies, Santa Clara, CA, USA), equipped with a 30 m × 0.25 mm ID, fused silica capillary column, with a 0.25 μM TG-5MS stationary phase (Thermo Fisher Scientific, Waltham, MA, USA). The injector and transfer line temperatures were 250 °C and 280 °C, respectively. The gas flow rate through the column was 1 mL/min. The column initial temperature was kept at 60 °C for 3 min, then increased to 140 °C at a rate of 7 °C/min, held at 140 °C for 4 min, increased to 300 °C at a rate of 5 °C/min and kept for 1 min. Identification of metabolites was performed using the standard NIST 08, and GMD mass spectra libraries and, when available, by comparison with analytical standards.

Peak detection and retention time correction were carried out with the R library XCMS and parameters used for peak deconvolution were manually optimized [71]. The resulting matrices were processed using an in-house Python script and a total area normalization was performed to compensate for sample dilution biases [72].

### 4.7. Striatal DA and DOPAC Assessment

Striatal tissue was sonicated in 0.25 mL of 0.2 M perchloric acid and then centrifuged at 9391× *g* for 15 min at 4 °C. The supernatant was transferred and filtered in Spin-X centrifuge tube filters (0.45 μm). The filtrate was diluted 1:10. Twenty microlitres were injected into an HPLC apparatus, equipped with a reverse-phase column (LC-18-DB, 15 cm, 5 μm particle size; Supelco, Milano, Italy) and a coulometric detector (ESA Coulochem II, Chelmsford, MA US) to quantify DA and DOPAC. Electrodes were set at +150 mV (oxidation) and 250 mV (reduction). The mobile phase (nM composition was: NaH_2_PO_4_, 100; NA_2_EDTA, 0.1; n-octyl sodium sulphate, 0.5; 7.5% methanol; pH 5.5) was pumped (Jasco Europe, Italy) at 1 mL⁄min flow rate. The assay sensitivity for DA and DOPAC was 10 fmol/sample.

### 4.8. Statistical Analysis

Multivariate statistical analysis was performed on GC-MS data by using SIMCA-P software (ver. 15.0, Umetrics, Umeå, Sweden) [73]. Firstly, the variables were UV scaled and then PCA analysis was conducted, with the aim to explore sample distributions and to identify potential outliers (DmodX and Hotelling’s T^2^ tests were applied for this purpose).

Supervised analysis was subsequently used. PLS-DA was employed to observe the effect of the oligomers on the samples compared to vehicles. For each model, the variance and the predictive ability (R^2^X, R^2^Y, Q^2^) were evaluatedand additionally and a permutation test (*n* = 300) was performed. The scores from each PLS-DA model were subjected to a CV-ANOVA to test for significance (*p* < 0.05). To study a possible linear relationship between a matrix Y (dependent variables, for example, the concentration of the DA and the DOPAC) and a matrix X (predictor variables, e.g., metabolites) a partial least squares regression (PLS) model was performed [74].

The variables responsible of the separation of the different classes of rats were extracted by the loading plot from the PLS-DA models. Significant changes of the variables concentration were tested through the U-Mann Whitney test followed by Holm-Bonferroni correction for multiple comparisons (GraphPad Prism software (version 7.01, GraphPad Software, Inc., San Diego, CA, USA) Finally, once the significant metabolites were selected, a Spearman correlation was performed between each selected significant metabolite and the levels of the DA and DOPAC.

## 5. Conclusions

The search for biomarkers of diagnosis and disease progression has been an area of intense investigation in PD. Compared to hypothesis-driven research, the untargeted metabolomics approach we used offers a great advantage to simultaneously measure different compounds, allowing identification of disease-specific alterations in individual compounds or metabolic pathways. Our analysis adds evidence supporting the role of αSyn oligomers in the aetiology of PD, and suggests that intracerebral HαSynO infusion is a valid preclinical model useful to identify new therapeutic targets and candidate biomarkers distinguishing a healthy condition from PD.

## Figures and Tables

**Table 1:** Summary of the statistical parameters of the supervised models of the mesencephalon analysis.

Multivariate Analysis					
Mesencephalon					
Models	R^2^X	R^2^Y	Q^2^	*p*-Value	Permutation Test: Intercept R^2^/Q^2^
**Veh R vs. Veh L**	0.38	0.82	−0.03	ns	-
**Oligo R vs. Oligo L**	0.55	0.84	0.57	0.04	0.56/−0.20
**Veh vs. Oligo (all)**	0.45	0.64	−0.20	-	-
**Veh R vs. Oligo R**	0.39	0.72	−0.07	-	-
**Veh L vs. Oligo L**	0.77	0.83	0.72	0.01	0.41/−0.21

ns = not significant; - = not available.

**Table 2:** Summary of the results of the univariate statistical analysis and Spearman correlation of the mesencephalon. Univariate statistical analysis and Spearman correlation of mesencephalic tissue from the left hemisphere of rats treated with Vehicle or HαSynOs + and − indicate respectively an increase or decrease of the metabolite in the OLIGO as compared with the Vehicle group.

Mesencephalon					
Vehicles L vs. Oligomers L					
METABOLITES	OLIGO	*p*-Value	*p*-Value Corrected	Spearman Correlation	
				Dopamine	
				R^2^	*p*-Value
**Dehydroascorbic acid**	−	0.008	0.004	0.7	0.01
**Glycine**	−	0.03	0.04	0.55	0.05
**Myo-Inositol**	−	0.02	0.04	0.6	0.04
**Threonine**	+	0.04	0.04	−0.01	0.8

+ = increased; − = decreased.

**Table 3:** Summary of the results of the univariate statistical analysis of the serum samples. Univariate statistical analysis of serum from rats treated with Vehicle or HαSynOs. + and - indicate respectively an increase or decrease of the metabolite in the OLIGO as compared with the Vehicle group.

Serum			
Vehicles vs. Oligomers			
METABOLITES	OLIGO	*p*-Value	*p*-Value Corrected
Fructose	+	0.05	0.06
Glycine	−	0.008	0.02
Mannose	+	0.05	0.06
Urea	−	0.03	0.06

+ = increased; − = decreased.
